# Functional cooperation of *spns2* and *fibronectin* in cardiac and lower jaw development

**DOI:** 10.1242/bio.20134994

**Published:** 2013-06-20

**Authors:** Yu Hisano, Satoshi Ota, Shinji Takada, Atsuo Kawahara

**Affiliations:** 1 Laboratory for Cardiovascular Molecular Dynamics, Riken Quantitative Biology Center, Furuedai 6-2-3, Suita, Osaka 565-0874, Japan; 2 Okazaki Institute for Integrative Bioscience, National Institute of Natural Sciences, Okazaki, Aichi 444-8787, Japan; 3 Department of Basic Biology, The Graduate University for Advanced Studies (SOKENDAI), Okazaki, Aichi 444-8787, Japan

**Keywords:** Sphingosine-1-phosphate, *spns2*, *fibronectin*, Jaw, Heart

## Abstract

The lipid mediator sphingosine-1-phosphate (S1P) is a regulator of cardiac development in zebrafish, as disruption of its receptor *s1pr2* or transporter *spns2* causes migration defects in cardiac progenitors. To examine the genetic interaction of S1P signaling and the cell adhesion molecule fibronectin, we have established a *fn;spns2* double mutant. Cardiac migration defects in *fn;spns2* mutants were more severe than those in *fn* or *spns2* mutants. We further found that the lower jaw morphology was disorganized in the *fn;spns2* mutant, while it had a slightly shortened anterior–posterior distance in the ventral pharyngeal arch in *fn* and *spns2* mutants relative to wild type. Knockdown of *fn* in the *s1pr2* mutant, but not in the *s1pr1* mutant, resulted in severe defects in cardiac migration and ventral pharyngeal arch arrangement. Further, in the background of the *fn* mutant, knockdown of *endothelin receptor A* (*ednra*), which was downregulated in the *spns2* mutant, caused pharyngeal defects resembling those in the *fn;spns2* mutant. These results strongly suggest that Spns2-S1PR2 signaling and fibronectin cooperatively regulate both cardiac and lower jaw development in zebrafish.

## Introduction

The bioactive lipid mediator sphingosine-1-phosphate (S1P) plays important roles in various types of biological processes including angiogenesis, inflammation and immunity (Skoura and Hla, 2009; Spiegel and Milstien, 2011; Hisano et al., 2012). In zebrafish, S1P is involved in cardiac development by regulating the myocardial migration, as evidenced by the migration defects of cardiac progenitors in an S1P receptor (S1PR) and an S1P transporter mutant, the *s1pr2* and *spns2* mutants (Kupperman et al., 2000; Osborne et al., 2008; Kawahara et al., 2009). S1PRs consist of at least five G protein-couple receptors (S1PR1–S1PR5) that show differential expression patterns during mouse embryogenesis (Ohuchi et al., 2008; Meng and Lee, 2009). Intercellular S1P signaling through S1PRs activates various downstream signaling pathways (Takuwa et al., 2012), leading to diverse cellular responses including cell proliferation, differentiation and cell migration. However, the developmental function of S1P signaling through the S1P-S1PR axis remains largely unclear.

The cell adhesion molecule fibronectin is a major component of the extracellular matrix (ECM) and is involved in various cellular processes including cytoskeletal organization and cell migration (Wierzbicka-Patynowski and Schwarzbauer, 2003). The *fibronectin* (*fn*) mutant, which shows a loss of fibronectin function in zebrafish, has defective boundary formation in its anterior somites (Koshida et al., 2005), suggesting fibronectin contributes to the epithelialization of somites. Additionally, treatment of anti-fibronectin antibody in chick embryos inhibits myocardial migration (Linask and Lash, 1986). In mice, myocardial specification is normally observed in *fibronectin* knockout mice, whereas myocardial migration is inhibited (George et al., 1997). It has also been shown that fibronectin is required for adherens junction formation between cardiac progenitors in zebrafish (Trinh and Stainier, 2004). These studies all suggest an important role for fibronectin in vertebrate cardiac development. Nevertheless, there is much uncertainty on how fibronectin cooperates with other signaling molecule(s) when regulating cardiac development and other organogenesis.

In this study, we established a double mutant, *fn;spns2*, to investigate the genetic interaction of fibronectin and S1P signaling in zebrafish. We found the two separated hearts phenotype in the *fn;spns2* double mutant was more severe than that in either *fn* mutant or *spns2* mutants. Further, the anterior–posterior distance of the lower jaw was shorter in the *fn* and *spns2* mutants, while the ventral pharyngeal arch structure was significantly impaired in the *fn;spns2* double mutant. Our results genetically reveal a functional cooperation between S1P signaling and fibronectin for the regulation of myocardial migration and lower jaw formation.

## Results and Discussion

### Cardiac progenitor migration regulated by Spns2 and fibronectin

**Fig. 1.**
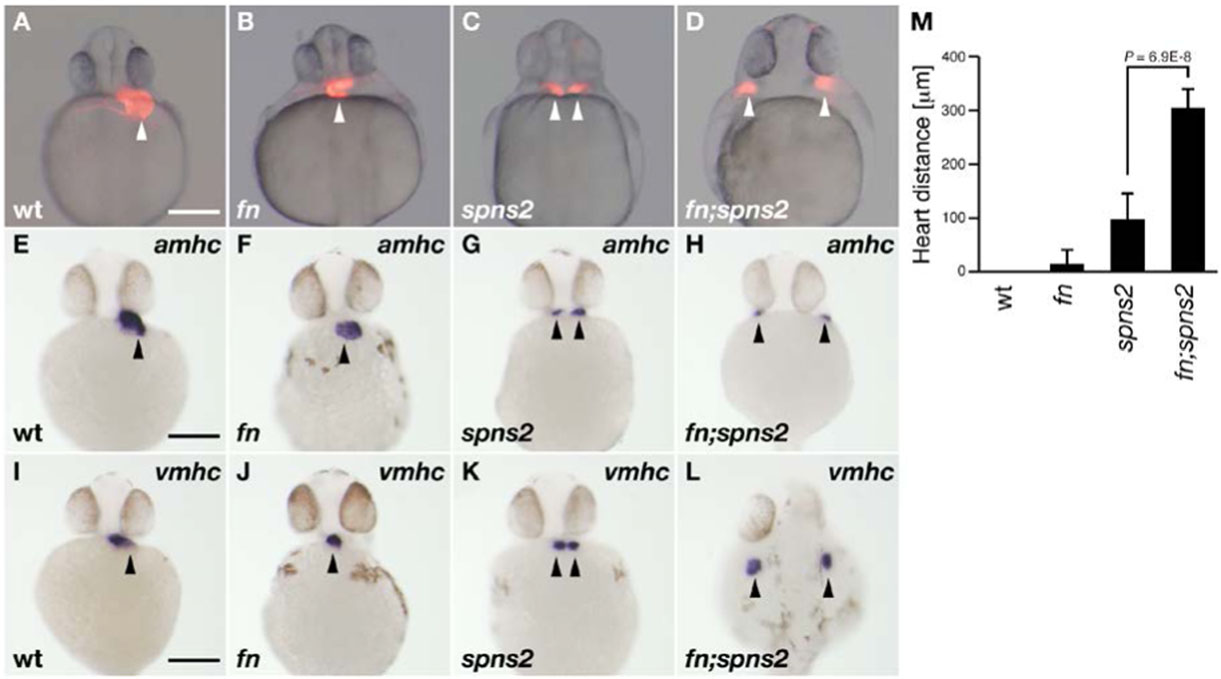
Cardiac morphology. Heart positions are indicated by the arrowheads. (**A–D**) Cardiac morphology visualized by mRFP expression derived from *Tg*(*cmlc2:mRFP*). All images show ventral views at 28 hpf. (**E–L**) Whole-mount *in situ* hybridization with *amhc* and *vmhc* RNA probes. All images show ventral views at 30 hpf except for panel L (dorsal view). Genotyping was performed by genomic sequencing after taking pictures, wt (A,E,I), *fn* mutant (B,F,J), *spns2* mutant (C,G,K) and *fn;spns2* double mutant (D,H,L). Scale bars: 200 µm. (**M**) Average distances between two hearts from multiple experiments; error bars represent standard deviations.

The heart tube develops from bilateral cardiac progenitors in the anterior lateral plate mesoderm in all vertebrate (Miura and Yelon, 2011) and laterally positioned cardiac progenitors coordinately move toward the midline and fuse to form the heart tube. In zebrafish, disrupting *s1pr2* (S1P receptor) or *spns2* (S1P transporter) results in defective migration of these cardiac progenitors and in cardia bifida (two separated hearts), indicating S1P signaling regulates myocardial migration (Kupperman et al., 2000; Osborne et al., 2008; Kawahara et al., 2009). It has been shown that the cell adhesion molecule fibronectin also contributes to the cardiac morphogenesis, as *fn* mutants partially penetrate the cardia bifida phenotype (Trinh and Stainier, 2004). Our established *fn^kt259^* mutant, a null mutant with a premature termination at codon 241, predominantly presented a straight heart tube phenotype (31/133 embryos obtained from the crossing of *fn^kt259^* heterozygous fish) at 25 hours post-fertilization (hpf), but also had a minor population showing cardia bifida (3/133 embryos), a low penetration result that may be explained by the genetic background. To examine the genetic interactions between S1P signaling and fibronectin, we generated double mutant zebrafish *fn^kt259^;spns2^ko157^* by the crossing *spns2^ko157^* and *fn^kt259^* mutants. As shown in Fig. 1, the *fn:spns2* double mutant displayed two widely separated hearts (19/322 embryos obtained from the crossing of *fn^kt259^;spns2^ko157^* heterozygous fish). The distances between hearts were much greater than those of the *spns2* (cardia bifida; 30/145 embryos obtained from the crossing of *spns2^ko157^* heterogeneous fish) or *fn* mutants (straight heart tube; 31/133 embryos obtained from the crossing of *fn^kt259^* heterozygous fish) (supplementary material Table S1). The genotypes of individual mutants were confirmed by the direct sequencing of individual *spns2* and *fn* genomic loci. Whole-mount *in situ* hybridization analysis revealed that the expressions of the cardiac differentiation markers *amhc* (*atrial myosin heavy chain*) and *vmhc* (*ventricular myosin heavy chain*) in *fn;spns2* mutant (*amhc*, *n* = 7; *vmhc*, *n* = 9) at 30 hpf were located at lateral distances greater than those of the *spns2* (*amhc*, *n* = 8; *vmhc*, *n* = 7) or *fn* mutants (*amhc*, *n* = 8; *vmhc*, *n* = 8) (Fig. 1). Further, we observed beating by the two separated hearts in the *fn;spns2* mutant (supplementary material Movies 1, 2). Thus, the cardiac progenitor migration, but not the cardiac differentiation is predominantly impaired in *fn;spns2* double mutants, suggesting that Spns2 and fibronectin synergize to promote cardiac progenitor migration.

### Lower jaw development is cooperatively regulated by Spns2 and fibronectin

**Fig. 2.**
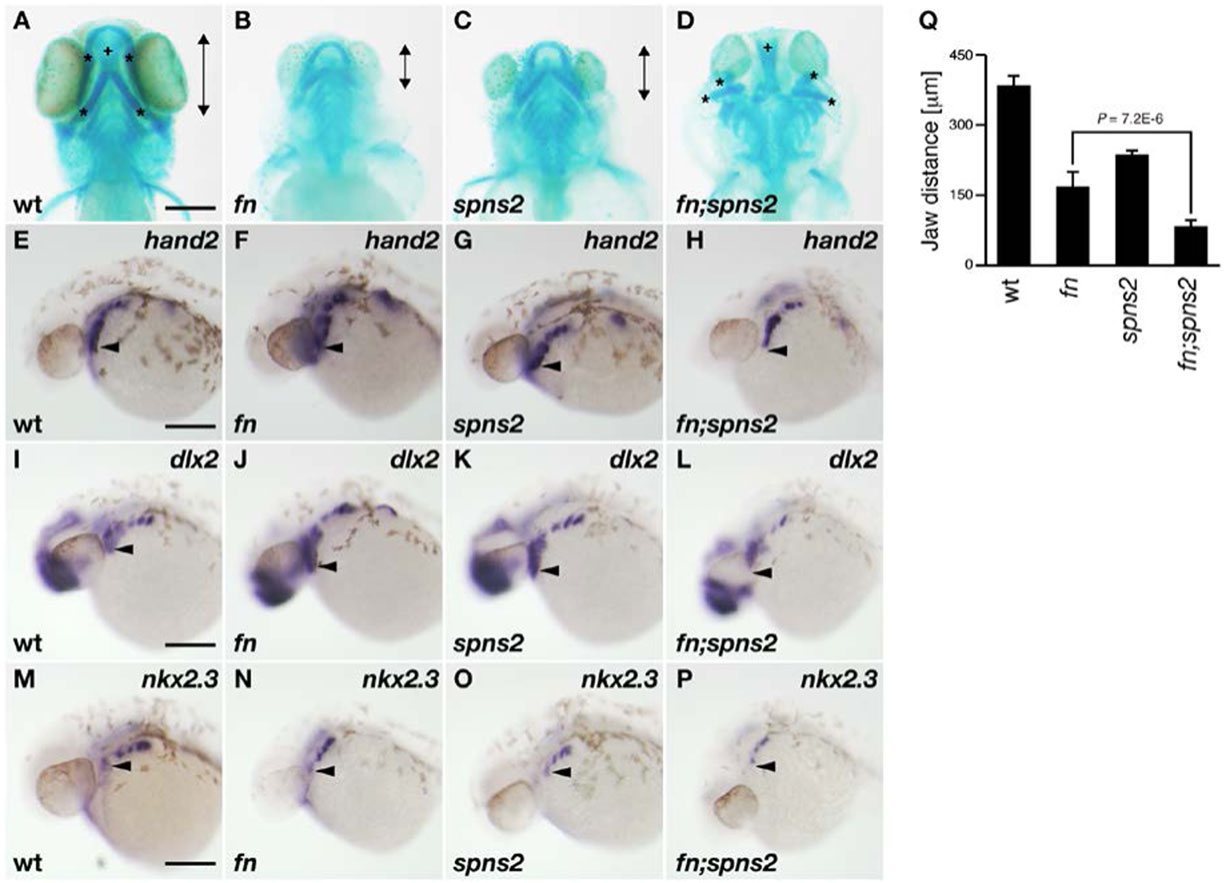
Lower jaw morphology. (**A–D**) Lower jaw morphology at 4 dpf was visualized by Alcian Blue staining (ventral view). Anterior–posterior distances of the ventral pharyngeal arch (*) is indicated by the length of the double-headed arrows. The dorsal pharyngeal structure is identified by the crosses (+). (**E–P**) Whole-mount *in situ* hybridization using *hand2*, *dlx2* and *nkx2.3* RNA probes. The anteroventral position of these markers is marked by the arrowheads. All images show lateral views at 30 hpf. Genotyping was performed by genomic sequencing after taking pictures. wt (A,E,I,M), *fn* mutant (B,F,J,N), *spns2* mutant (C,G,K,O), and *fn;spns2* double mutant (D,H,L,P). Scale bars: 200 µm. (**Q**) Average anterior–posterior distances of the ventral pharyngeal arch from multiple experiments; error bars represent standard deviations.

The facial skeleton is formed from mutual interactions between cranial neural crest cells and both the pharyngeal endoderm and ectoderm of zebrafish. Additionally, secreted proteins such as Endothelin1, BMPs and Fgfs are key regulators involved in the craniofacial development (Alexander et al., 2011; Yamauchi et al., 2011). It has been shown that the *spns2* mutant displays a disorganized anterior pharyngeal endoderm (Osborne et al., 2008). We noticed that our *fn;spns2* double mutant exhibits severe defects in ventral facial morphology. Therefore, we examined the pharyngeal arch structure of individual mutants by Alcian Blue staining at 4 days post-fertilization (dpf). The anterior–posterior distance of the ventral pharyngeal arches (Meckel's, palatoquadrate and ceratohyal cartilages) in the *spns2* or *fn* mutants was shorter than that of wild type (Fig. 2; supplementary material Table S2). On the other hand, the number and morphology of the ceratobranchial arch were relatively normal. The pharyngeal defects are consistent with a recent report that demonstrated morphological defects of the lower jaw in both *s1pr2* and *spns2* mutants (Balczerski et al., 2012). We also found that the cell adhesion molecule fibronectin is also required for the proper lower jaw development (Fig. 2; supplementary material Table S2). In clear contrast, the structure of the ventral pharyngeal arch (Fig. 2D, asterisks) was disorganized in the *fn;spns2* double mutant, whereas that of the dorsal pharyngeal structure (trabecular cartilage) appeared normal (Fig. 2D, cross). Jaw development is regulated by the cooperation of several transcriptional factors including the *hand2*, *dlx* and *nkx* family genes (Miller et al., 2003; Trinh et al., 2005; Talbot et al., 2010). Whole-mount *in situ* hybridization using the pharyngeal markers, *hand2*, *dlx2* and *nkx2.3* at 30 hpf revealed that their anteroventral expressions of *hand2*, *dlx2* and *nkx2.3* were reduced in *fn;spns2* mutants (*hand2*, *n* = 7; *dlx2*, *n* = 7; *nkx2.3*, *n* = 7) compared to the their posterior expressions, whereas the expression patterns and intensities of these markers were normal in *fn* mutants (*hand2*, *n* = 7; *dlx2*, *n* = 5; *nkx2.3*, *n* = 7) and *spns2* mutants (*hand2*, *n* = 7; *dlx2*, *n* = 8; *nkx2.3*, *n* = 8). The genotypes of individual mutants were confirmed by the direct sequencing of *spns2* and *fn* genomic loci after taking pictures. Because the first pouch endoderm is required for the pharyngeal arch formation (Alexander et al., 2011), our results suggest that Spns2 and fibronectin contribute to the formation of the ventral pharyngeal arch by regulating the anteroventral expression of various pharyngeal markers.

### S1PR2, but not S1PR1, cooperates with fibronectin in cardiac and lower jaw development

**Fig. 3.**
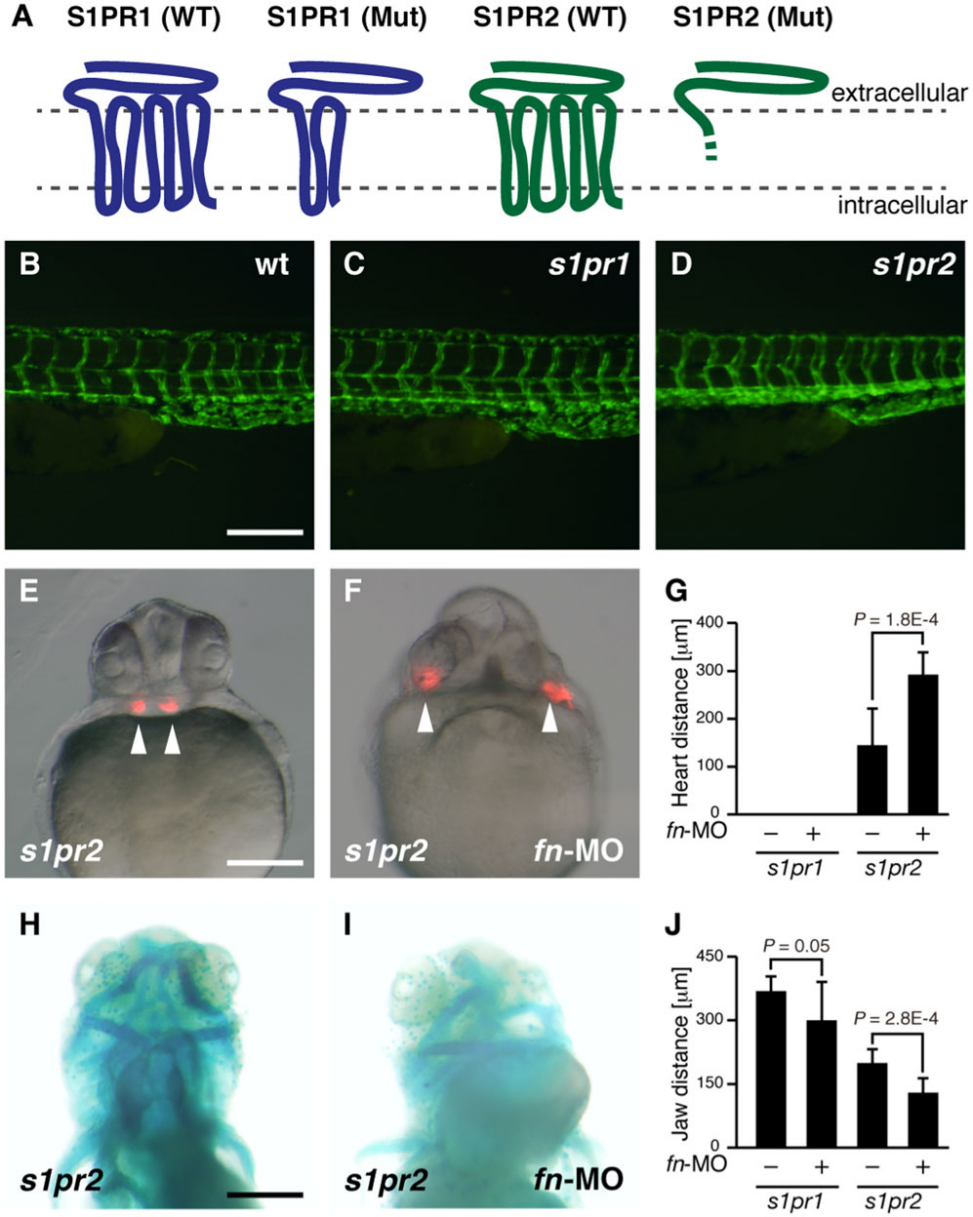
Knockdown phenotype of *fibronectin* in S1PRs mutant. (**A**) Membrane topology of S1P receptors and their mutants. The region of frameshift-mediated amino acids compared with the S1PR2 wild type (WT) is shown by the dashed line. (**B**,**C**,**D**) Fluorescence microscopy of intersegmental vessels of wt (B), *s1pr1* mutant (C) and *s1pr2* mutnat (D) at 2 dpf. Endothelial cells are visualized by EGFP expression derived from *Tg*(*fli1a:EGFP*). (**E**,**F**) Cardiac morphology visualized by mRFP expression derived from *Tg*(*cmlc2:mRFP*). All images show ventral views at 28 hpf. (**G**) Average distances between two hearts from multiple experiments; error bars represent standard deviations. (**H**,**I**) Lower jaw morphology at 4 dpf was visualized by Alcian Blue staining (ventral view). (**J**) Average anterior–posterior distances of the ventral pharyngeal arch from multiple experiments; error bars represent standard deviations. Genotyping was performed by genomic sequencing or heteroduplex mobility assays after taking pictures. Scale bars: 200 µm.

Spns2 functions as an S1P transporter (Kawahara et al., 2009), suggesting that some S1PRs contribute to cardiac and jaw development. The *s1pr2* mutant has been found to present the cardia bifida phenotype in zebrafish (Kupperman et al., 2000), whereas three independent groups recently reported that the knockdown of *s1pr1* causes severe defects in cardiac and vascular development (Gaengel et al., 2012; Tobia et al., 2012; Mendelson et al., 2013), with the circulation of blood cells being particularly impaired in *s1pr1*-depleted embryos. We therefore examined the functional interaction of S1PR1/2 and fibronectin. Using TALEN (transcription activator-like effector nuclease) technology, we recently established *s1pr1* and *s1pr2* knockout fish (Hisano et al., 2013; Ota et al., 2013). We confirmed that *s1pr2* knockout mutants show cardia bifida phenotype (Fig. 3E). However, no obvious cardiac or vascular defects in *s1pr1* knockout mutants were seen during early embryogenesis (Fig. 3C; supplementary material Movies 3, 4). Thus, we conclude that zygotic *s1pr1* mutants showed normal blood circulation and intersegmental vessel angiogenesis, which disagrees with the aforementioned reports. Those studies all used identical S1PR1-morpholino. It is possible that the S1PR1-morpholino caused off-target effects or also affected the maternal message of *s1pr1*. To explain the different conclusions, it would be best to study a maternal-zygotic *s1pr1* mutant, which will be available in a future study. When Fn-MO (10 ng) was injected into the *s1pr2* mutant, a more severe cardia bifida phenotype was induced compared to the *s1pr2* mutant (Fig. 3E–G). Consistent with these results, severe defects in cardiac migration and lower jaw morphology were observed when S1PR2-MO (10 ng) was injected into *fn* mutants (supplemental material Fig. S1), which agrees with previous knockdown analysis that used S1PR2-MO and Fn-MO (Matsui et al., 2007). Such a cooperative cardiac defect was not observed in *s1pr1* embryos injected with Fn-MO (supplementary material Table S1), which instead showed a straight heart tube phenotype similar to that in *fn* mutants. Fn-MO-injected *s1pr1* mutants embryos had a slightly shorter anterior–posterior distance in their ventral pharyngeal arch structure, quite unlike the disorganized ventral pharyngeal arch structure observed in Fn-MO-injected *s1pr2* mutants (Fig. 3H–J; supplementary material Table S2). Additionally, severe defects in cardiac migration and lower jaw morphology were observed when S1PR2-MO (10 ng) was injected into *fn* mutants (supplemental material Fig. S1). These results suggest that S1PR2, but not S1PR1, cooperates with fibronectin in both cardiac and lower jaw development. It has been reported that both *s1pr2* and *spns2* mutants lack the anterior endoderm (Balczerski et al., 2012). Because signaling pathways through the Spns2-S1PR2 axis regulate cell proliferation of the anterior endoderm tissue and therefore affect the positioning of the ventral pharyngeal arch, further analysis will be required to clarify how S1P signaling and fibronectin together control the movement and adhesion of anterior pharyngeal endodermal cells.

### Endothelin receptor A, a possible mediator downstream of Spns2-S1PR2 signaling

**Fig. 4.**
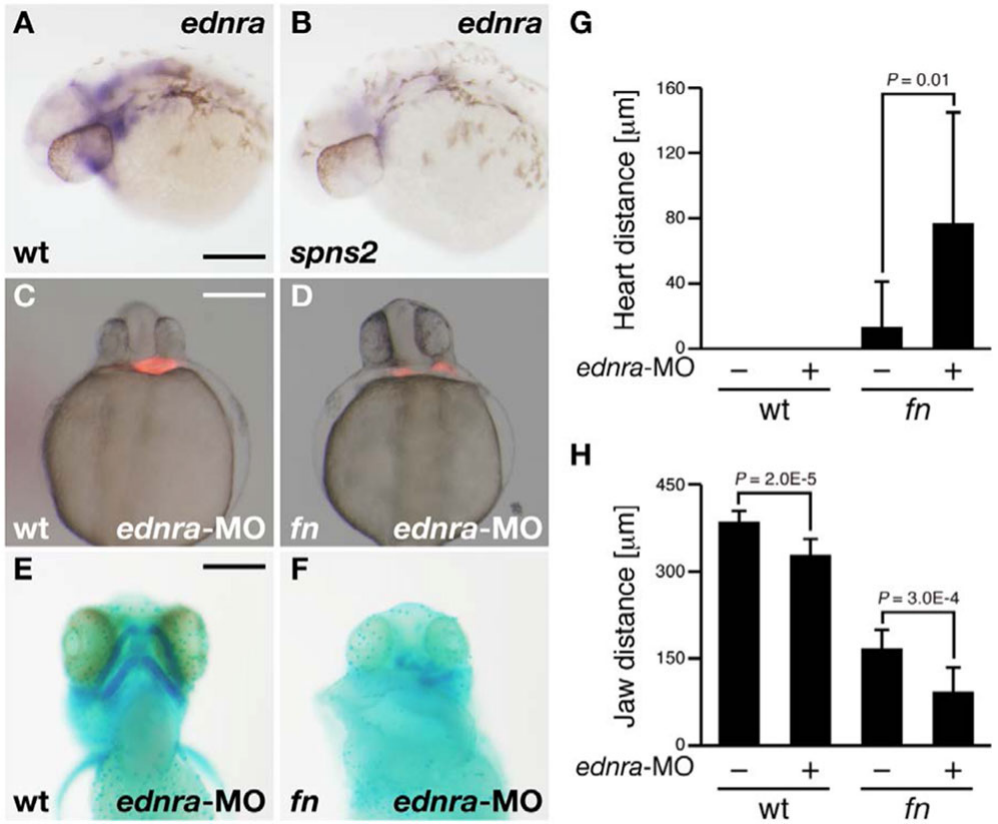
Knockdown phenotype of *endothelin receptor A* (*ednra*) in *fn* mutants. (**A**,**B**) Whole-mount *in situ* hybridization using the *ednra* RNA probe. The expression of *ednra* was suppressed in the *spns2* mutant. Both images show lateral views at 30 hpf. (**C**,**D**) Cardiac morphology visualized by mRFP expression derived from *Tg*(*cmlc2:mRFP*). Both images show ventral views at 28 hpf. (**E**,**F**) Lower jaw morphology at 4 dpf was visualized by Alcian Blue staining (ventral view). Genotyping was performed by genomic sequencing after taking pictures. wt (A,C,E), *spns2* mutant (B) and *fn* mutant (D,F). Scale bars: 200 µm. (**G**,**H**) Average distances between hearts (G) and anterior–posterior distances of the ventral pharyngeal arch (H) from multiple experiments; error bars represent standard deviations.

Both Spns2 and S1PR2 are involved in cardiac and lower jaw development. To identify the genes regulated by the Spns2-S1PR2 axis, we performed microarray analysis. Total RNA was isolated from uninjected, S1PR2-MO (10 ng)-injected and Spns2-MO (10 ng)-injected embryos at 25 hpf, and their gene expression profiles were compared. We found that the expression of *endothelin receptor A* (*ednra*) was downregulated in both Spns2-depleted and S1PR2-depleted embryos (see Materials and Methods). Consistent with this result, the expression of *ednra* in the pharyngeal arches of the *spns2* mutant (*n* = 10) was reduced compared to that of wild type (*n* = 7) (Fig. 4A,B). In both zebrafish and mouse, disruption of *endothelin1* (*edn1*) causes a loss or transformation of the lower jaw (Nair et al., 2007; Tavares et al., 2012). Because Edn1 functions through its cognate type-A receptor Ednra, Ednra can be considered a key regulator in pharyngeal development (Nair et al., 2007). However, it is not clear how Ednra cooperates with other molecule(s) during jaw development. Therefore, we investigated the functional interaction between Ednra and fibronectin. Heart morphology seemed normal when Ednra-MO (10 ng) was injected into wild-type embryos (*n* = 13), while cardiac defects in Ednra-MO injected *fn* embryos (*n* = 11) were slightly more severe than those of *fn* embryos (Fig. 4C,D; supplementary material Table S1). Further, a disorganization of the ventral pharyngeal arch arrangement in Ednra-MO injected *fn* embryos (*n* = 10) was observed relative to that of Ednra-MO injected wild-type embryo (*n* = 7) (Fig. 4E,F; supplementary material Table S2). The genotypes of individual mutants were confirmed by the direct sequencing of *spns2* and *fn* genomic loci after taking pictures. Although in a cell culture system Edn1 increases the adhesion of amelanotic melanocytes to fibronectin (Ma et al., 2006), how Edn1 signaling affects fibronectin function remains unclear. Further analysis will be required to determine whether Edn1 regulates the fibronectin-mediated cellular interaction during the ventral pharyngeal arch arrangement. One clue comes from both S1P-S1PR2 and End1-Ednra being critical in the patterning of the ventral pharyngeal arch, which agrees with our demonstrating that Ednra, a possible downstream target of the Spns2-S1PR2 axis, synergizes with fibronectin to promote the lower jaw development.

### Conclusion

In this study, using zebrafish genetic mutants (*fn*, *spns2* and *fn;spns2*), we demonstrated that Spns2-S1PR2 signaling and the cell adhesion molecule fibronectin cooperatively regulate the migration of cardiac progenitors. Further, Spns2-S1PR2 and fibronectin synergize to promote ventral pharyngeal cartilage formation. Because the expression of *ednra* in the pharyngeal arches of *spns2* mutants is reduced, we propose that Ednra contributes to the lower jaw arrangement by cooperating with fibronectin.

## Materials and Methods

### Zebrafish mutants

Mutant alleles of *fibronectin^kt259^* (*fn^kt259^*) and *spns2^ko157^* were used (Koshida et al., 2005; Kawahara et al., 2009). To obtain double mutants, the *spns2^ko157^* allele was crossed into the *fn^kt259^* allele. Embryos of *fn^kt259^;spns2^ko157^* were obtained from *fn^kt259^;spns2^ko157^* heterogeneous fish. Embryos of *spns2^ko157^* and *fn^kt259^* were obtained from *fn^kt259^* and *spns2^ko157^* heterogeneous fish, respectively. Genotyping of *spns2^ko157^* and *fn^kt259^* single mutants and *fn^kt259^;spns2^ko157^* double mutants was performed by direct sequencing of individual genomic loci as described below. To monitor the cardiac development, the transgenic line *Tg*(*cmlc2:mRFP*) was used.

### Establishment of *s1pr1* or *s1pr2*-knockout zebrafish

TALEN constructs targeting *s1pr1* or *s1pr2* were described previously (Hisano et al., 2013; Ota et al., 2013). TALEN mRNAs (400 pg each) were injected into blastomeres at the 1–2 cell stage of zebrafish embryos. Identification of potential F0 founders and F1 embryos having mutant allele was performed by HMA (heteroduplex mobility assay) (Ota et al., 2013). The *s1pr1^ko311^* allele was deleted from +362 to +371 of the *s1pr1* coding region, while the *s1pr2^ko322^* allele was deleted from +179 to +188 of the *s1pr2* coding region. To monitor the cardiac and vascular development, the transgenic lines *Tg(cmlc2:mRFP)* and *Tg(fli1a:EGFP)* were used. *s1pr1* or *s1pr2* mutant embryos were obtained by the crossing individual F1 heterogeneous fish.

### Preparation of genomic DNA and genotyping of *spns2* and *fn* mutants

Genomic DNA was isolated using the Gentra Puregene Tissue Kit (Qiagen) according to the manufacturer's protocol. For the genotyping of the *spns2* mutant, the *spns2* genomic locus was amplified by PCR using the following primers: spns2-S, 5′-TCAAGGAATGTGAGCCATGT-3′; and spns2-AS, 5′-GGATGCCAGGTAGAAGACA-3′. For genotyping of the *fn* mutant, the *fn* genomic locus was amplified by PCR using the following primers: fn-S, 5′-CTTACTCAAGCTTAACTGG-3′; and fn-AS, 5′-ACCAAGACTAGTAGTGTGCAG-3′. In the case of embryos stained with Alcian Blue or analyzed by whole-mount *in situ* hybridization, nested PCR was performed using the following primers: fn-S2, 5′-GGTTCTAATGGGAAACATCTGC-3′; and fn-AS2, 5′-GAGAAGCATGCCTCTCACAC-3′. The genotypes of *spns2* and *fn* were determined by direct sequencing of the PCR amplified fragments.

### RNA probes and whole-mount *in situ* hybridization

Antisense RNA labeled with digoxigenin (DIG) was prepared using the RNA labeling kit (Roche). Whole-mount *in situ* hybridization was performed as previously described (Hanaoka et al., 2006). Briefly, embryos were hybridized with DIG-labeled anti-sense RNA probes at 65°C overnight in hybridization buffer (50% formamide, 5× SSC, 5 mM EDTA, 0.1% Tween 20, 50 µg/ml heparin and 1 mg/ml RNA torula). Then, embryos were washed twice at 65°C for 30 min with washing buffer I (50% formamide, 2× SSC and 0.1% Tween 20), twice at 65°C for 30 min with washing buffer II (2× SSC and 0.1% Tween 20), twice at 65°C for 30 min with washing buffer III (0.2× SSC and 0.1% Tween 20) and once at room temperature for 15 min with maleic acid buffer (0.1 M maleic acid [pH 7.5]). Embryos were incubated with anti-DIG alkaline phosphatase (Roche) in blocking buffer (0.1 M maleic acid [pH 7.5], 5% sheep serum and 2% blocking reagent) for 4 hr. After embryos were washed with phosphate buffer saline (PBS) containing 0.1% Tween 20 (PBST), color reactions were performed using BM purple (Roche) as the substrate.

### Alcian Blue staining

Embryos at 4 dpf were fixed overnight by 4% paraformaldehyde in PBST. Embryos were washed with acid alcohol buffer (0.37% HCl and 70% ethanol) and incubated with 0.1% Alcian Blue (Sigma) in acid alcohol buffer. After 3 times washing with acid alcohol buffer, embryos were incubated for 10 min with bleaching buffer (1% H_2_O_2_ and 1% KOH).

### Knockdown analysis using antisense morpholinos and microarray analysis

Antisense morpholinos for *fibronectin*, *spns2*, *s1pr2* and *ednra* were obtained from Gene Tools as follows: Fn-MO, 5′-TTTTTTCACAGGTGCGATTGAACAC-3′; Ednra-MO, 5′- AGTGGTGTGTTCACCTGTTTGAGGT-3′; Spns2-MO, 5′-GGAGGGAATATGTGATGCTTACTTC-3′; and S1PR2-MO, 5′-CCGCAAACAGACGGCAAGTAGTCAT-3′ (Trinh and Stainier, 2004; Nair et al., 2007). Individual morpholinos (10 ng) were injected into the yolk of 1–4 cell stage embryos. Total RNA was isolated with TRIzol reagent (Invitrogen) from individual embryos at 25 hpf. Microarray analysis was performed using a zebrafish-specific Affymetrix chip (15617 zebrafish probes). The expression of *ednra* was downregulated in Spns2-MO-injected (Log2 Ratio: −1.06) and S1PR2-MO-injected embryos (Log2 Ratio: −1.06).

## Supplementary Material

Supplementary Material
