# Supplementary material for: Functional cooperation of spns2 and fibronectin in cardiac and lower jaw development
Source: Biol Open. 2013 Jun 20;2(8):789–94. doi: 10.1242/bio.20134994 (PMC3744070; doi:10.1242/bio.20134994)
Supplement: Supplementary Material [file supp_2_8_789__index.html]

Functional cooperation of spns2 and fibronectin in cardiac and lower jaw development — Functional cooperation of spns2 and fibronectin in cardiac and lower jaw development — Supplementary Material 

# Functional cooperation of *spns2* and *fibronectin* in cardiac and lower jaw development

## 

**Files in this Data Supplement:**

- Supplementary Material - Yu Hisano et al. doi: 10.1242/bio.20134994
- Movie 1 - **Movie 1. Beating heart in a wt embryo at 28 hpf.**
- Movie 2 - **Movie 2. Beating heart in a *fn-spns2* double mutant embryo at 28 hpf.**
- Movie 3 - **Movie 3. Blood circulation in a wt embryo at 28 hpf.**
- Movie 4 - **Movie 4. Blood circulation in an *s1pr1* mutant embryo at 28 hpf.**
