# Supplementary material for: Functional cooperation of spns2 and fibronectin in cardiac and lower jaw development
Source: Biol Open. 2013 Jun 20;2(8):789–94. doi: 10.1242/bio.20134994 (PMC3744070; doi:10.1242/bio.20134994)
Supplement: Supplementary Material [file supp_bio.20134994_bio.20134994-s1.pdf]

Supplementary Material

Yu Hisano et al. doi: 10.1242/bio.20134994

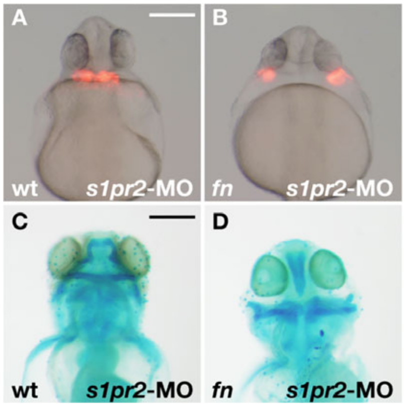

Fig. S1. Knockdown phenotype of S1PR2 in *fn* mutant. (**A**,**B**) Cardiac morphology visualized by mRFP expression derived from *Tg*(*cmlc2:mRFP*). Both images show ventral views at 28 hpf. (**C**,**D**) Lower jaw morphology at 4 dpf was visualized by Alcian Blue staining (ventral view). Genotyping was performed by genomic sequencing after taking pictures. wt (A,C) and *fn* mutant (B,D). Scale bars: 200 µm.

Table S1. Heart morphology.

	MO	Heart distance (µm)	sd	n
WT		0.00	0.00	10
Fn		13.20	27.84	10
S1PR1		0.00	0.00	6
S1PR2		145.56	78.39	7
spns2		97.24	50.01	11
Fn-spns2		304.43	37.81	7
WT	fn	3.11	10.78	12
S1PR1	fn	0.00	0.00	7
S1PR2	fn	294.37	46.38	10
WT	ednra	0.00	0.00	13
Fn	ednra	58.11	49.42	11

Table S2. Lower jaw morphology.

	MO	Jaw distance (µm)	sd	n
WT		385.97	20.56	16
Fn		169.49	32.73	11
S1PR1		371.16	32.43	8
S1PR2		200.89	33.08	11
spns2		240.28	10.06	6
Fn-spns2		84.00	13.43	8
WT	fn	247.90	88.75	10
S1PR1	fn	299.70	92.76	13
S1PR2	fn	131.84	32.02	8
WT	ednra	330.24	26.58	7
Fn	ednra	95.29	43.98	10

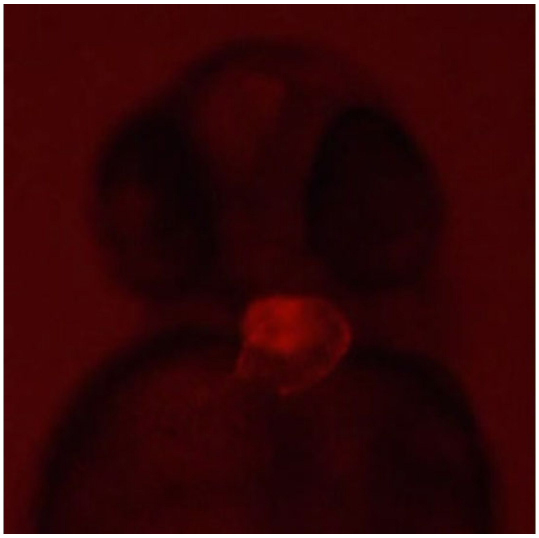

Movie 1. Beating heart in a wt embryo at 28 hpf.

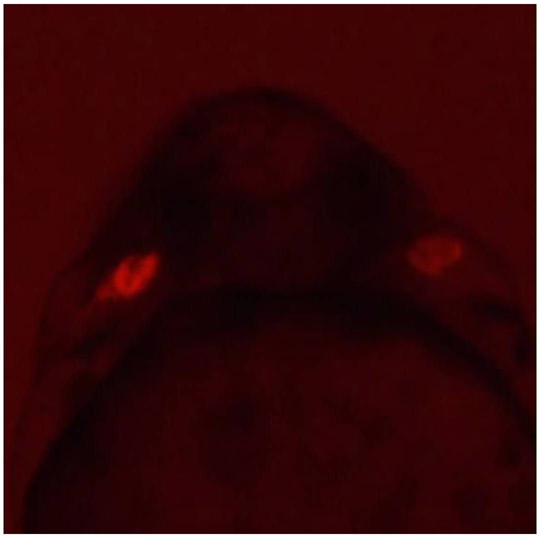

Movie 2. Beating heart in a *fn-spns2* double mutant embryo at 28 hpf.

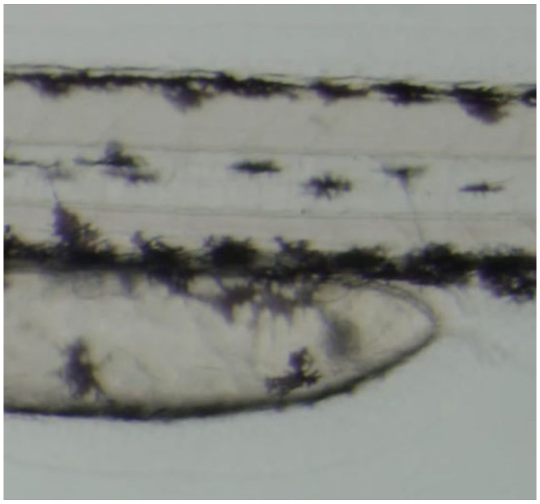

Movie 3. Blood circulation in a wt embryo at 28 hpf.

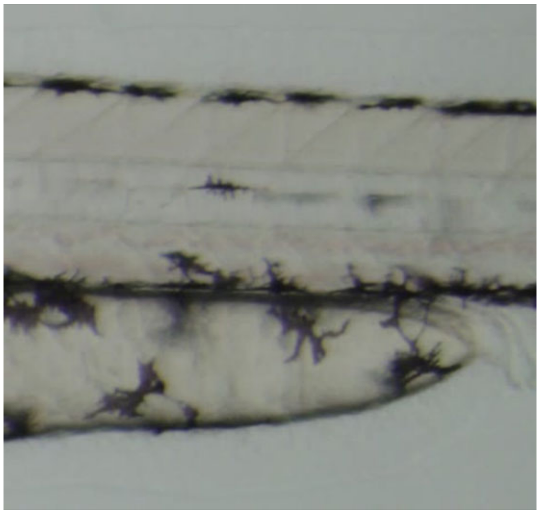

Movie 4. Blood circulation in an *s1pr1* mutant embryo at 28 hpf.
